# Mid- and Long-Term Results of Endovascular Treatment in Thoracic Aorta Blunt Trauma

**DOI:** 10.1100/2012/396873

**Published:** 2012-05-03

**Authors:** Luigi Irace, Antonella Laurito, Salvatore Venosi, Francesco Giosuè Irace, Alban Malay, Bruno Gossetti, Luciano Bresadola, Roberto Gattuso, Ombretta Martinelli

**Affiliations:** Department of Vascular Surgery, “Sapienza” University of Rome, 00161 Rome, Italy

## Abstract

*Study Aim*. Evaluation of results in blunt injury of the thoracic aorta (BAI) endovascular treatment. *Materials and Methods*. Sixteen patients were treated for BAI. Thirteen patients had associated polytrauma, 4 of these had a serious hypotensive status and 4 had an hemothorax. In the remaining 3, two had a post-traumatic false aneurysm of the isthmus and 1 had a segmental dissection. In those 13 patients a periaortic hematoma was associated to hemothorax in 4. All patients were submitted to an endovascular treatment, in two cases the subclavian artery ostium was intentionally covered. *Results*. One patient died for disseminated intravascular coagulation. No paraplegia was recorded. No ischemic complications were observed. A type I endoleak was treated by an adjunctive cuff. During the followup (1–9 years) 3 patients were lost. A good patency and no endoleaks were observed in all cases. One infolding and 1 migration of the endografts were corrected by an adjunctive cuff. *Conclusion*. The medium and long term results of the endovascular treatment of BAI are encouraging with a low incidence rate of mortality and complications. More suitable endo-suite and endografts could be a crucial point for the further improvement of these results.

## 1. Introduction

Blunt traumatic thoracic aortic injury is associated with a high mortality rate and has been implicated as the second most common cause of death in trauma patients, behind only intracranial hemorrhage. Moreover, the association with multiple trauma is often coexisting in these patients, and this produces a buildup of comorbidity. Motor vehicle accidents were responsible for 96.7% of the injuries, and blunt trauma, due to down fall, caused the remaining 3.3% [1, 2].

It has been estimated that less than 25% of patients with such an injury live to be evaluated in a hospital, and, of those who do, up to 50% will die within 24 hours. Conventional surgical repair typically involves a high posterolateral thoracotomy with or without cardiopulmonary bypass and significant blood loss, which can negatively impact the pulmonary, cardiac, and neurologic status of the patient. Historically, open repair of traumatic aortic injuries has been associated with a 28% mortality rate and a 16% paraplegia rate [3, 4].

Thoracic endovascular aortic repair (TEVAR) is a rapidly evolving therapy in the treatment of a variety of thoracic aortic pathologies. TEVAR involves placing an endovascular stent graft into the thoracic aorta from a remote peripheral location under imaging guidance. TEVAR offers the potential for a durable aortic repair while avoiding the morbidity of a thoracotomy, aortic cross clamping, and cardiopulmonary bypass. Obviously, stroke, spinal cord ischemia, and other complications that are associated with open repair can also occur with TEVAR. Nevertheless, this new approach seems to have the rationale for lowering mortality and complications in thoracic aortic injuries [5, 6].

## 2. Materials and Methods

From January 2001 to January 2011, sixteen patients, 13 males and 3 females, were admitted and treated in our department for traumatic injuries of the thoracic aorta. The patients' age ranged from 20 to 69, and in the majority (13/16) motor vehicle accidents were responsible for the trauma while in two of the remaining an accidental downfall and in one an attempted suicide.

Thirteen patients came to hospital in critical condition; 11 of these presented a periaortic hematoma with hemothorax in 4, while in the remaining 2 an intimal tear was associated to intramural hematoma. In the last 3 patients, with an history of an old trauma, a pseudoaneurysm was present in two and a focal dissection in one.

In all the cases a TC scan with an interval slice of 1 millimeters was carried out to evaluate site and extension of the thoracic aorta lesions and the possible coexisting injuries of organs and systems.

A total body computed tomography angiography (CTA) was always carried out in order to define the aortic lesion and to detect potential concomitant injuries. CT scan allowed to obtain accurate aortic measures to evaluate the feasibility of the endovascular endografting in terms of distance from supraaortic trunks, arch angulation, proximal and distal landing zone, and the caliber of iliac-femoral axis. The preinterventional measurement by CT of aortic diameter at the proximal and distal landing zones ranged from 20.7 ± 1.2 mm and 2.1 ± 1.9 mm, respectively. The aortic arch angle ranged between 83° and 126° with an acute angle (<92°) were observed in 3 cases. Arteriography and transesophageal echocardiography were never performed before TEVAR.

The attention was focused on the lesion site, on measurement of vessel diameter, lesion length, distance between the subclavian artery and lesion itself, and on aortic arch angulation and morphology and diameters of iliac and femoral vessels.

**Figure 1:**
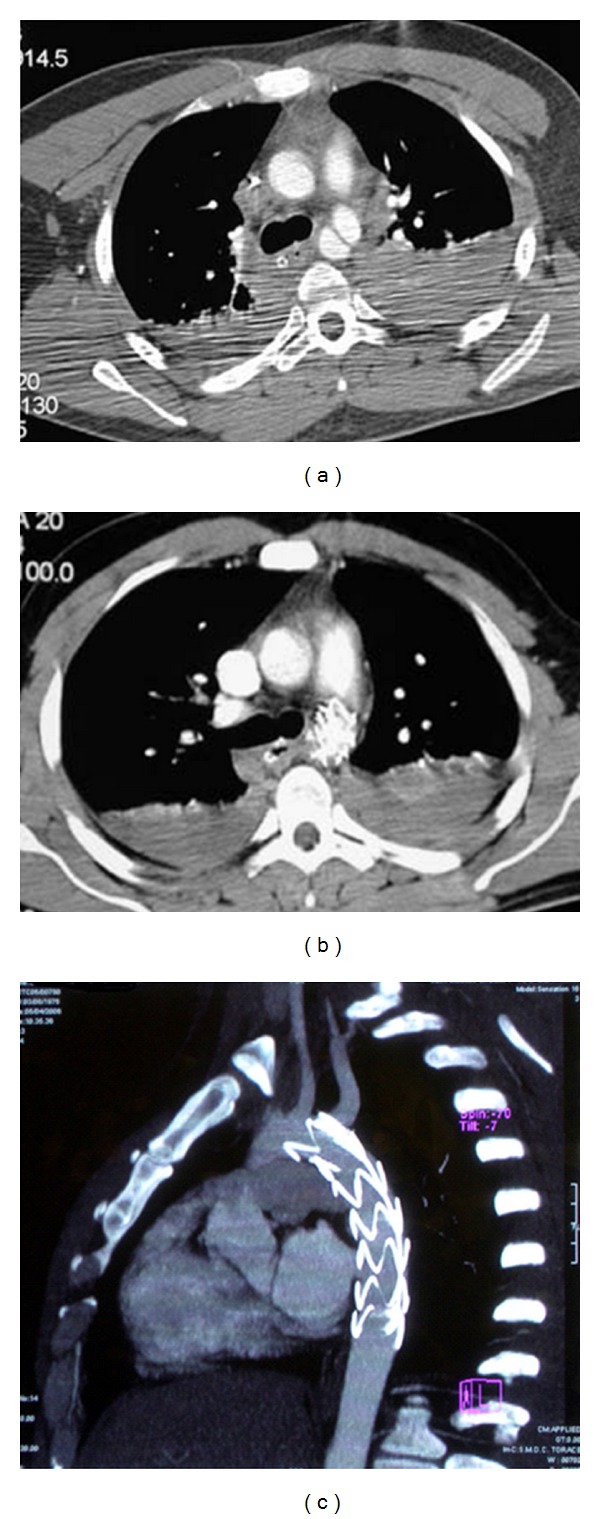
24 y. Patient-motorcycle accident. (a) Preoperative CT scan. (b) Postoperative CT scan. (c) CT scan control at 7 years.

In all cases an endovascular procedure was carried out; the endograft was released just below the subclavian artery origin in 14 cases (Figure 1) while in 2 the vessel ostium was intentionally covered without ischemic symptoms in postoperative period and during followup.

The prosthesis was inserted by a surgical exposure of the femoral artery in the majority of cases (14/16) while an iliac access was preferred in 2 for the small vessel diameter.

In relation to the emergency conditions and to the small segment to cover, no cerebrospinal fluid drainage and no heparin treatment was applied during surgery in 13, while in the remaining 3, with a chronic history, heparin was given at the standard dosage.

All patients were operated upon general anesthesia and the implanted endografts were Medtronic Talent 1, Medtronic Valiant 6, Medtronic Captiva 7, and Gore Tag 2.

During the all procedures a controlled hypotension (90 mm/hg) was maintained to avoid the endograft dislodgement during the release and a final angiography was carried out to evaluate the correct graft position and the endoleak absence.

During the followup (1–9 years), a TC scan was carried out in all patients before the discharge and thereafter every year.

In 9 patients some adjunctive procedures were carried out: 2 splenectomies performed before the endovascular procedure; 4 bone stabilization and 3 thoracic drainage, all after the endograft deployment.

## 3. Results

During the postoperative period and before the discharge, 3 major complications were collected: one patient died for a DIC, another developed, in the third postoperative day, a cerebral ischemia with a right haemiplegia totally regressed in few days, and in the last one a type I endoleak was diagnosed by CT scan. In this case immediately a reintervention with the deployment of an adjunctive endograft was carried out with a good final result.

No paraplegia was recorded, and in the 2 cases, in whom the coverage of the subclavian artery origin was needed, no clinical signs of ischemia were detected also if, by means of Duplex scan examination, a subclavian steal syndrome was detected.

**Figure 2:**
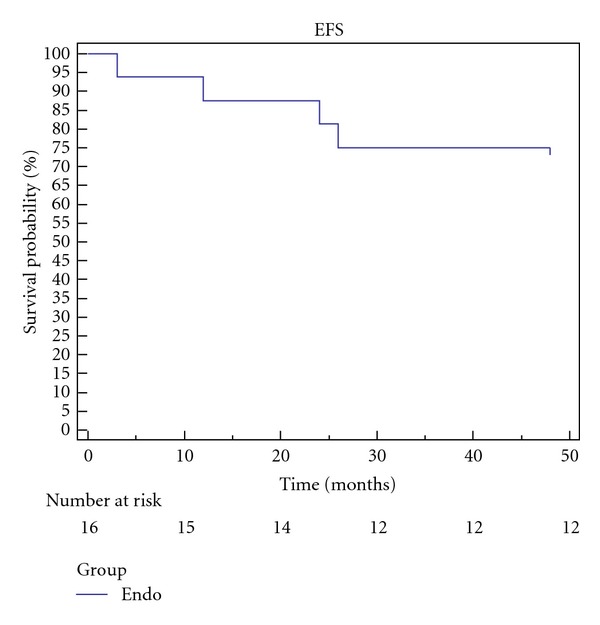
The Kaplan-Meier curve for event free survival (EFS). Patients lost to the follow-up were censored (*n* = 3).

During the followup, 3 patients were lost and the remaining 13 were followed for a period ranging from 1 to 9 years; 5 patients had a followup longer than 5 years. A survival curve describing the Kaplan-Meier overall survival with indication of the number of patients at risk at each time interval is showed in Figure 2. In this time the CT scan control revealed 1 infolding and 1 distal dislodgement, and, in both cases, the treatment consisted in the positioning of an adjunctive endograft.

## 4. Discussion and Conclusions

The standard treatment of patients with traumatic injuries of the thoracic aorta has been early surgery with interposition graft. Recently the new developments in resuscitation changed the medical approach to these patients because lowering the blood pressure and reduction of the systolic dynamics give the opportunity to stabilize the patient and to delay the surgical repair. Nevertheless, the high risk of dramatic rupture and the high incidence rate of perioperative mortality let to search new less invasive and early approach like the endovascular one [7–9].

In a recent review endorsed by the American Society for Vascular Surgery, in which 7768 patients were collected, the mortality and morbidity rate in patients submitted to endovascular treatment for thoracic aortic injuries was dramatically lower (9%) in comparison to those treated surgically (19%) or medically (46%) with a *P* < 0.01. Also the risk of spinal cord ischemia (SCI) and of end-stage renal disease (ESRD) has showed a higher incidence in the surgical group (resp., 9% and 8%) in comparison to endovascular one (resp., 3% and 5%) with a *P* = 0.01, like also graft and pulmonary infection [5, 10].

Nevertheless, in our opinion some arising problem relative to the endovascular treatment remain unsolved [10, 11].

First of all the correct sizing of the aorta in a patient with hypovolemia, haemodynamic collapse, early resuscitation and young. The choice of a not adequate graft let to more 10% rate of early complication as migration or collapse. These data are confirmed by the Second American Association for the Surgery of Trauma (AAST) that reported an alarmingly high risk (20%) of device-related complications and included 18 patients (14%) with endoleak treated with repeat TEVAR [9] or endograft explantation and open descending thoracic aorta repair (DTAR) [6]. Although the anatomic lesion in trauma is very often limited in a short segment, no one has known what is the fate of the thoracic aorta especially in young patients in whom a 1 cm increase is reached between 20 and 80 years. The long-term results are not known due to a few large reports in the literature. The fate of thoracic endograft positioned in a young patient, in a small aortic size, is not clear. In our experience 5 patients were followed in 5 years or more and 2 complications were detected in both cases treated and solved by endovascular reintervention.

Concerning the use of intraoperative anticoagulation, our rationale is to avoid heparin in acute patients in whom other traumas are associated and limit its use only in stable or chronic conditions.

The low incidence rate of spinal cord ischemia (no case in our experience) is due probably to the short segment covered by the endograft: generally the distal part of the aortic arch or the proximal part of the descending aorta. So the critical zone that includes the segment between T6 and L1 is rarely involved by the procedure. For this reason no spinal cord drainage was carried out in our series. Moreover, the temporary interruption of perfusion to the spinal cord related to aortic cross-clamping during open repair and systemic hypotension that may complicate thoracotomy have been implicated as factors responsible for the higher incidence of spinal cord ischemia as compared with TEVAR.

The last point of this approach is the coverage of the left subclavian artery (LSA). Our experience is similar to other authors, and it is not easy in emergency to carry out a correct evaluation of the Willis circulation and of the dominant vertebral artery; furthermore, in all the cases when the LSA is mandatorily covered, an angiography must be performed to visualize either the posterior circulation or the LSA revascularization by the vertebral artery. Generally there is no need in deploying a plug, because there is no an aneurysmal pathology and the lesion is not atherosclerotic in most cases.

In conclusion, also in our experience the endovascular approach to thoracic aorta trauma may appear to favor endograft repair though a number of issues are still unresolved: poor conformation to the arch, frequent need to cover the left subclavian artery, unknown natural history of the repair in the young, optimal follow-up strategy, timing of repair, and the need for intraoperative anticoagulation in the setting of polytrauma. Another unsolved problem in young is the need of frequent control and radiation exposure. Probably new materials and new grafts' design are needed to reduce mid- and long-term complications' rate. Consequently, some authors suggest to treat surgically young patients and with TEVAR old patients and those not fit for surgery for comorbidities.
